# Supplementary material for: Unraveling the Antibacterial Mechanism of Plasma-Activated Lactic Acid against Pseudomonas ludensis by Untargeted Metabolomics
Source: Foods. 2023 Apr 10;12(8):1605. doi: 10.3390/foods12081605 (PMC10137701; doi:10.3390/foods12081605)
Supplement: Supplementary file 1 [file foods-12-01605-s001.zip › foods-2285911-supplementary.pdf]

foods

Supplementary material

Unraveling the antibacterial mechanism of Plasma-activated lactic acid against *Pseudomonas ludensis* by untargeted metabolomics

Zhaobin Wang[1], Xiaoting Wang[2], Xiaowei Sheng[1], Luling Zhao[1], Jing Qian[1], Jianhao Zhang[1,*] and Jin Wang[3,*]

[1] National Center of Meat Quality and Safety Control, Collaborative Innovation Center of Meat Production and Processing, Quality and Safety Control, College of Food Science and Technology, Nanjing Agricultural University, Nanjing 210095, China
[2] College (School) of Food and Drug, Luoyang Normal University, Luoyang 471934, China
[3] Key Laboratory of Environmental Medicine and Engineering, Ministry of Education, and Department of Nutrition and Food Hygiene, School of Public Health, Southeast University, Nanjing 210009, China

[1,*] Correspondence: nau_zjh@njau.edu.cn

[3,*] Correspondence: jin.wang6@mail.mcgill.ca

Table S1. Differential metabolites of PALA treated *P. lundensis* identified through untargeted metabolomics

Serial number	Name	Log2 (T/CK)	*P*-value	VIP
1	3-[4-(3,5-dichloro-4-pyridinyl) piperazino]-1,1,1-trifluoro-2-propanol	-3.08	1.45E-05	1.0053
2	Maltopentaose	-6	5.00E-09	1.9066
3	ϒ-Glutamylcysteine	-8.06	9.58E-08	2.5691
4	Adenosine	-3.53	8.65E-06	1.1434
5	L-Homocitrulline	-5.91	2.47E-07	1.8878
6	Xanthosine	-6.54	9.26E-06	2.1513
7	Reduced nicotinamide adenine dinucleotide	-5.56	1.36E-06	1.7552
8	D-α-Hydroxyglutaric acid	-7.76	2.53E-07	2.4907
9	Gamma-Glu-Leu	-5.7	1.41E-06	1.8345
10	D-Ribose	-3.84	5.28E-08	1.2234
11	S-Adenosylhomocysteine	-7.52	5.44E-05	2.4119
12	N-Acetylaspartic acid	-8.23	1.33E-09	2.6149
13	Ranolazine	-5.94	1.43E-06	1.9073
14	D-(-)-Fructose	-4.74	4.52E-09	1.5041
15	2-Methylbutyric acid	2.2	0.013751	1.0636
16	Inosine	-3.49	6.19E-06	1.1224
17	N6-Succinyl Adenosine	-5.87	6.85E-09	1.8689
18	Nicotinamide adenine dinucleotide (NAD+)	-5.02	4.14E-06	1.6248
19	Nitrofurantoin	-6.12	2.14E-06	1.956
20	Dl-Glyceraldehyde3-phosphate	-3.56	5.67E-07	1.134
21	Retrorsine	-5.03	8.21E-07	1.5988
22	L (+)-Ornithine	-4.91	1.05E-06	1.5739
23	Uridine	-5.12	1.68E-05	1.6898
24	N6-Acetyl-L-lysine	-8.77	1.51E-08	2.7963
25	Pantothenic acid	-6.67	3.07E-07	2.1411
26	(R)-3-Hydroxy myristic acid	-3.87	0.000631	1.3559
27	DL-Norvaline DL	-3.09	9.76E-05	1.0253
28	2'-Deoxycytidine	-4.72	4.98E-06	1.5255
29	2-(acetylamino)-3-(1H-indol-3-yl) propanoic acid	-7.79	1.31E-10	2.474

30	7-methylpyrimido[4,5-d] pyrimidin-4-amine	-4.83	4.38E-06	1.5551
31	L-(+)-Citrulline	-4.48	7.65E-06	1.4475
32	2'-Deoxyinosine	-8.86	9.98E-07	2.8932
33	4-Butylresorcinol	-6.82	5.73E-07	2.1732
34	Cyclic ADP-ribose	-4.88	8.48E-08	1.5576
35	N-Acetylglycine	-5.09	1.38E-05	1.6626
36	2-Isopropylmalic acid	-6.8	1.01E-09	2.1624
37	N-Acetylornithine	-7.67	5.72E-07	2.4661
38	Asp-glu	-4.74	7.36E-07	1.4946
39	Citric acid	-6.04	3.15E-09	1.9228
40	L-Glutathione oxidized	-6.04	4.44E-06	1.9646
41	L-Hydroxyproline	-3.81	3.25E-07	1.2094
42	mesaconic acid	-4.75	1.22E-08	1.5114
43	(2E)-1-(2-hydroxy-3,4,5,6-tetramethoxy-phenyl)-3-phenylprop-2-en-1-one	5.26	3.40E-05	1.7199
44	6-Methylquinoline	-3.88	6.66E-05	1.2836
45	L-Glutathione (reduced)	-8.77	3.67E-07	2.8232
46	N-{6-[(7-chloro-4-quinazolinyl) oxy]-3-pyridi-nyl}-4-methoxybenzamide	-3.7	2.19E-05	1.1619
47	UMP	-5.19	9.72E-07	1.6665
48	Cytidine 5'-monophosphate (hydrate)	-5.18	3.67E-06	1.673
49	N-Acetyl-L-tyrosine	-5.25	2.86E-10	1.669
50	2-mercapto-4-(2-thienyl)-5,6,7,8-tetrahydro-quinoline-3-carbonitrile	-4.51	1.22E-05	1.4531
51	D-glutamine	-3.65	2.46E-06	1.1675
52	Neopterin	-5.5	4.72E-07	1.7624
53	Adenosine diphosphate (ADP)	-4.11	2.85E-06	1.3052
54	4-morpholino-3-nitrobenzene-1-sulfonamide	-4.94	2.56E-05	1.5952
55	N-[2-(1,5-dimethyl-4-nitro-1H-pyrazol-3-yl) vi-nyl] -N,N-dimethylamine	-5.58	1.67E-06	1.7998
56	Epinephrine	-4.4	1.01E-06	1.3889
57	D-2-Aminoadipic acid	-5.19	1.21E-09	1.6471
58	5'-S-Methyl-5'-thioadenosine	-4.57	7.93E-08	1.4557
59	NAD+	-4.23	4.92E-05	1.3695
60	4-cyclopropyl-6-methoxy-1,3,5-triazin-2-amine	-4.44	1.96E-05	1.4491
61	RLH	2.93	0.003268	1.1264
62	Epigallocatechin	-5.35	1.52E-06	1.7208
63	Gedunin	-5.59	1.79E-06	1.779
64	gamma-Glutamylcysteine	-5.43	6.02E-06	1.752
65	2,4-Dihydroxybenzoic acid	-8.43	5.61E-10	2.6771
66	N1-(1H-1,2,3-benzotriazol-5-yl)-3,4,5-tri-methoxybenzamide	3.51	3.17E-05	1.1014
67	4-chloro-5-morpholino-2-quinoxalin-2-ylpyri-dazin-3(2H)-one	-3.68	2.44E-05	1.1823
68	Guanosine	-3.39	1.23E-06	1.0827
69	13,14-Dihydro-15-keto-tetranor prostaglandin F1α	-3.02	0.000786	1.0617
70	Adenylocuccinic Acid	-4.85	3.26E-07	1.5371

71	Cytidine 5'-Monophosphate-N-Acetylneuraminic Acid	-4.9	8.21E-06	1.559
72	FKK	-4.79	7.97E-06	1.5097
73	Glycyl-L-leucine	-5.05	9.48E-07	1.6217
74	α-Lapachone	-5.12	2.49E-06	1.638
75	1-(4-methylphenyl)-3-(2-pyridylthio) pyrrolidine-2,5-dione	-4.39	1.16E-07	1.3933
76	S-Adenosyl-L-methionine	-3.78	7.33E-08	1.1988
77	ϒ-L-Glutamyl-L-glutamic acid	-4.58	6.81E-07	1.4647
78	2-{[methyl(2,3,4,5,6-pentahydroxyhexyl) amino] methylidene} malononitrile	-4.52	1.90E-06	1.4438
79	HKK	-3.87	8.55E-08	1.2272
80	S-Lactoyglutathione	-3.42	0.000129	1.1203
81	5-Hydroxytryptophan	-4.34	1.78E-06	1.3935
82	N8-Acetylspermidine	-4.77	3.63E-06	1.533
83	[4-(1H-indol-4-yl)piperazino](2-thienyl)methanone	-6.48	2.14E-10	2.0592
84	Uridine monophosphate (UMP)	-5.73	8.31E-08	1.814
85	L-Glutamic acid	-4.37	2.34E-06	1.3952
86	Kynurenic acid O-hexside	-3.96	3.73E-07	1.2516
87	S-(Methyl)Glutathione	-7.51	1.51E-06	2.4253
88	Hypoxanthine	-7.72	2.23E-09	2.4503
89	N,N-dimethyl-5-nitro-6-[3-(trifluoromethyl)phenoxy]pyrimidin-4-amine	4.85	3.78E-05	1.493
90	N-Acetyl-DL-glutamic acid	-5.64	2.76E-06	1.8091
91	5-Methyluridine	-3.4	8.07E-07	1.0869
92	L-arginine	-4.05	4.42E-07	1.2794
93	3-hydroxy-N-(1-hydroxy-4-methylpentan-2-yl)-5-oxo-6-phenylhexanamide	4.7	9.53E-06	1.5178
94	Tiglic acid	-3.71	3.35E-05	1.2001
95	2'-Deoxyadenosine 5'-monophosphate	-3.66	2.92E-06	1.1691
96	L-Cysteine-glutathione gisulfide	-3.59	9.21E-06	1.1598
97	2'-Deoxyadenosine 5'-monophosphate (dAMP)	-5.65	5.51E-09	1.7961
98	2-(Formylamino)Benzoic Acid	-4.54	1.40E-05	1.4761
99	3-(5-phenyl-1,3-oxazol-2-yl)-4-(trifluoromethyl)pyridine	-4.52	1.59E-06	1.4441
100	3-Methyladipic acid	-3.19	0.000116	1.053
101	nor-6β-Oxycodol	-4.49	7.60E-08	1.4331
102	Ala-Gln	-5.35	1.42E-06	1.7093
103	Ethylmalonate	-7.57	8.77E-10	2.4063
104	Xanthine	-4.37	0.00028	1.5461
105	Thymidine	-8.14	1.54E-06	2.6583
106	8-Bromoguanosine	-4.81	3.24E-06	1.519
107	Isophorone	-3.3	3.17E-06	1.0419
108	DL-Tryptophan	-4.38	3.72E-05	1.4448
109	Cytosine	-6.94	7.71E-08	2.2138
110	Maltotetraose	-7.12	8.78E-08	2.2668
111	2'-Deoxyadenosine	-4.77	1.02E-07	1.5077
112	L-Tyrosine	-3.79	8.31E-05	1.2667

113	gamma-Glutamylleucine	-4.84	7.24E-05	1.5964
114	N-Acetyl-aspartic acid	-5.78	5.71E-07	1.8523
115	2,6-Dihydroxypurine	-6.34	7.69E-07	2.0227
116	2-Hydroxycinnamic acid	-3.79	3.79E-06	1.223
117	Adenosine 5'-monophosphate	-5.8	2.68E-06	1.8729
118	N-Acetyl-L-leucine	-5.35	6.34E-05	1.6743
119	2-Furoic acid	-4.27	5.63E-07	1.3476
120	Guanosine monophosphate	-4.67	8.94E-06	1.5159
121	D-Raffinose	-5.12	0.000112	1.752
122	N-Acetylvaline	-5.13	6.84E-09	1.6306
123	2-Aminoadipic acid	-6.46	1.88E-08	2.0549
124	Cys-Gly	-7.05	1.49E-07	2.2527
125	4-(trifluoromethyl)nicotinic acid	-5.48	5.16E-06	1.755
126	D - (+)-Maltose	-6.41	8.05E-06	2.1003
127	Spermidine	-4.31	0.000136	1.4722
128	4-hydroxy-1-methyl-3-(phenylthio)-1,2-dihydroquinolin-2-one	-6.07	1.66E-08	1.9343
129	Pantetheine	-8.58	8.84E-09	2.7184
130	2'-Deoxyadenosine-5'-monophosphate	-5.02	2.27E-06	1.6129
131	Nicotinamide adenine dinucleotide	-5.12	7.33E-08	1.6314

Note: Name, name of metabolites; Log2 (T/CK), Log2 values of the ratio of average intensities of 4 replicates of treatment and control; *P*-value, statistical differences of two groups through t-test. VIP: Variable Importance in the Projection by the use of PLS-DA model.
